# Family Health Strategy Team’s Understanding of HIV: Actor-Network Theory

**DOI:** 10.1590/0034-7167-2025-0313

**Published:** 2026-08-21

**Authors:** Morgana Cristina Leôncio de Lima, Mônica Alice Santos da Silva, Clarissa Mourão Pinho, Luciana da Rocha Cabral, Erika Simone Galvão Pinto, Tatiane Gomes Guedes, Jael Maria de Aquino, Maria Sandra Andrade

**Affiliations:** I Universidade de Pernambuco. Recife, Pernambuco, Brazil; II Universidade Federal do Rio Grande do Norte. Natal, Rio Grande do Norte, Brazil; III Universidade Federal de Pernambuco. Recife, Pernambuco, Brazil

**Keywords:** HIV Seropositivity, Primary Health Care, Comprehensive Health Care, National Health Strategies, Public Health Nursing., Seropositividad para VIH, Atención Primaria de Salud, Estrategias de Salud Nacionales, Enfermería en Salud Pública.

## Abstract

**Objectives:**

to understand health care for people living with HIV from the perspective of Family Health Strategy professionals, in light of Actor-Network Theory.

**Methods:**

qualitative study with Family Health Strategy professionals in Recife, Brazil. Data were collected between October 2022 and April 2023 through semi-structured interviews. Analysis was conducted with the support of IRAMUTEQ software using Descending Hierarchical Classification.

**Results:**

six thematic categories emerged: training mediations-capacity-building as an actor in qualifying care; weakened support networks-the role of pharmaceutical services; Primary Health Care as the coordinating axis of networked care; controversies in continued care-fear, confidentiality, and exposure; and activation of health promotion, prevention, and surveillance within the care network.

**Final Considerations:**

challenges include universalizing rapid testing, resistance from some professional groups to new responsibilities, maintaining diagnostic confidentiality, and the persistence of stigma.

## RESUMEN

**Objetivos:**

comprender el cuidado en salud a personas que viven con VIH desde la perspectiva de profesionales de la Estrategia de Salud de la Familia, a la luz de la Teoría del Actor-Red.

**Métodos:**

estudio cualitativo realizado en Recife, Brasil. Datos recolectados entre octubre de 2022 y abril de 2023 mediante entrevistas semiestructuradas. El análisis se efectuó con IRAMUTEQ, utilizando Clasificación Jerárquica Descendente.

**Resultados:**

emergieron seis categorías: mediaciones formativas, con la capacitación como actor en la cualificación del cuidado; redes de apoyo fragilizadas, destacando la asistencia farmacéutica; la Atención Primaria como eje articulador del cuidado en red; controversias del cuidado continuado, atravesadas por miedo, sigilo y exposición; y activaciones de promoción, prevención y vigilancia en la red asistencial.

**Consideraciones Finales:**

los desafíos incluyen la universalización de la prueba rápida, la resistencia de algunos grupos profesionales a nuevas responsabilidades, el mantenimiento de la confidencialidad diagnóstica y la persistencia del estigma.

## INTRODUCTION

The decentralization of care for People Living with HIV (PLHIV) to Primary Health Care (PHC), from the perspective of health professionals from the core team of the Family Health Strategy (FHS) and grounded in Actor-Network Theory (ANT), reveals a complex process of reorganizing practices, knowledge, and relationships among multiple actors - human and non-human - that make up the care network^(1,2)^. The FHS team’s understanding shows that this movement is not limited to the incorporation of new responsibilities, but entails the reconfiguration of existing care networks, triggering instabilities, resistance, and negotiations that are inherent to any significant transformation in ways of providing care^(1,3)^.

Despite significant therapeutic advances, the number of new HIV infections continues to increase. In this context, strengthening the work of multiprofessional teams may promote a more effective approach to health promotion. From this perspective, in 2014, a national guideline was introduced to reorganize the care model for PLHIV, with the decentralization of services to Primary Health Care being considered a strategic element for improving care coordination^(4)^.

In this sense, it is fundamental to recognize the uniqueness, diversity, and specificities of each territory. The combination of knowledge from different professionals in building an articulated, expanded, and dialogical care approach points to a model of care based on the “living territory,” with an emphasis on the work of multidisciplinary teams and a community orientation^(5,6)^.

The articulation between human elements (professionals, users, managers) and non-human elements (rapid tests, physical infrastructure, protocols, information systems) is what enables - or weakens - the delivery of comprehensive care. Therefore, the success of decentralization will depend on the ability to recognize and mobilize all actors in the network, in order to promote dialogue, agreement-building, and shared responsibility^(2,7)^.

Advancing the consolidation of PHC as a legitimate setting for the care of People Living with HIV (PLHIV) requires more than normative guidelines; it demands attentive engagement, particularly through listening, with dynamic networks, sensitivity to emerging controversies, and investment in strengthening the system’s fragile connections. It is through this ongoing process of integrating knowledge, practices, and technologies that truly comprehensive and collaborative care is developed^(1,8)^.

## OBJECTIVES

To understand health care for people living with HIV from the perspective of Family Health Strategy professionals, in light of Actor-Network Theory.

## METHODS

### Ethical aspects

This study complied with the Guidelines and Standards for Research Involving Human Beings, in accordance with Resolution Nº. 466/12 of the Brazilian National Health Council (*Conselho Nacional de Saúde*-CNS). All participants signed the Informed Consent Form and the Voice Recording Authorization Form. Privacy and confidentiality of the collected data were strictly maintained. The study was approved in advance by the Research Ethics Committee under approval number 60697422.4.0000.5192.

In order to ensure participant anonymity, interviews were coded using an alphanumeric system in which statements were identified by the initials of the professional category-N (Nurse), NT (Nursing Technician), P (Physician), and CHW (Community Health Worker)-followed by sequential numbers.

### Theoretical and methodological framework

Data analysis was grounded in Bruno Latour’s Actor-Network Theory, which conceives phenomena as associations among human and non-human actors interconnected in networks^(1,8)^. This theoretical framework makes it possible to understand healthcare service flows, as well as the interactions and mediations that constitute practices, policies, and transformations within care networks^(2)^.

### Study design and methodological procedures

This is an exploratory study with a qualitative approach, aiming to understand the healthcare provided to people living with HIV (PLHIV) cared for in PHC. The study followed the Consolidated Criteria for Reporting Qualitative Research (COREQ).

### Study setting

The study was conducted within the PHC network of Recife, in the state of Pernambuco. The city is organized into eight Health Districts (HDs), with 133 Family Health Units and 281 Family Health teams^(9)^.

Regarding specialized municipal services for PLHIV, Recife has two Specialized Care Services (*Serviços de Assistência Especializada*-SAE): the first located at the Lessa de Andrade Polyclinic (HD IV) and the second at the Gouveia de Barros Polyclinic (HD I), which also houses the pediatric SAE and a Voluntary Counseling and Testing Center (*Centro de Testagem e Aconselhamento*-CTA)^(9)^. There is also a CTA at the Salomão Kelner Polyclinic (HD II). At the tertiary care level, the following facilities stand out: *Hospital Correia Picanço, Hospital das Clínicas* da UFPE, and *Hospital Universitário Oswaldo Cruz*.

### Study participants

Participants were healthcare professionals from the minimum staffing of the Family Health Strategy team, composed by a physician, a nurse, a nursing assistant or technician, and a Community Health Worker (CHW)^(10)^. The inclusion criteria were as follows: professionals of any gender, either permanent or contracted staff, physicians engaged in clinical care roles at the Family Health Units (FHUs) included in the study, and with at least one year of experience at the unit.

The exclusion criteria included professionals on medical, maternity, or other types of leave for more than 90 days. Unit selection was conducted by convenience sampling, and interviews were carried out at the healthcare facilities or in a location chosen by the participant. The number of interviews was determined according to the criterion of theoretical saturation, which was considered reached when no new information emerged within each professional category^(11)^. There was one refusal, justified by the participant’s lack of available time.

### Data collection and organization

Data were collected between October 2022 and April 2023 through individual semi-structured interviews, scheduled in advance by phone or in person. Interviews were conducted during participants’ working hours, in a private space within the health units.

The instrument used was an interview guide addressing objective and subjective aspects of PLHIV care, covering topics such as access, diagnosis, service structure, training, prevention actions, and health education.

### Study phases

Data analysis followed thematic content analysis^(11)^, structured in three phases: (1) pre-analysis, (2) material exploration, and (3) treatment of results and data interpretation. Interviews were transcribed verbatim for subsequent analysis^(11)^.

### Data analysis

The interview textual corpus was analyzed with the support of the software Interface de R pour les Analyses Multidimensionnelles de Textes et de Questionnaires (IRAMUTEQ), using the Descending Hierarchical Classification (DHC) technique. This technique organizes textual material based on lexical and conceptual proximity, generating dendrograms that illustrate the structure of thematic classes^(12-14)^.

Critical interpretation of the data was carried out in light of Actor-Network Theory^(1,8)^, which enables a reflective reading of the relationships and controversies identified in the process of decentralizing care for PLHIV.

## RESULTS

A total of 89 professionals from the FHUs participated in the study, including 25 nurses, 21 nursing technicians, 12 physicians, and 31 Community Health Workers. Overall, 73 participants (82%) were female, aged between 20 and 65 years, with a mean age of 44 years. Regarding marital status, 38 participants (42.7%) reported being single, a proportion very similar to that of married participants (41.6%).

**Figure 1:**
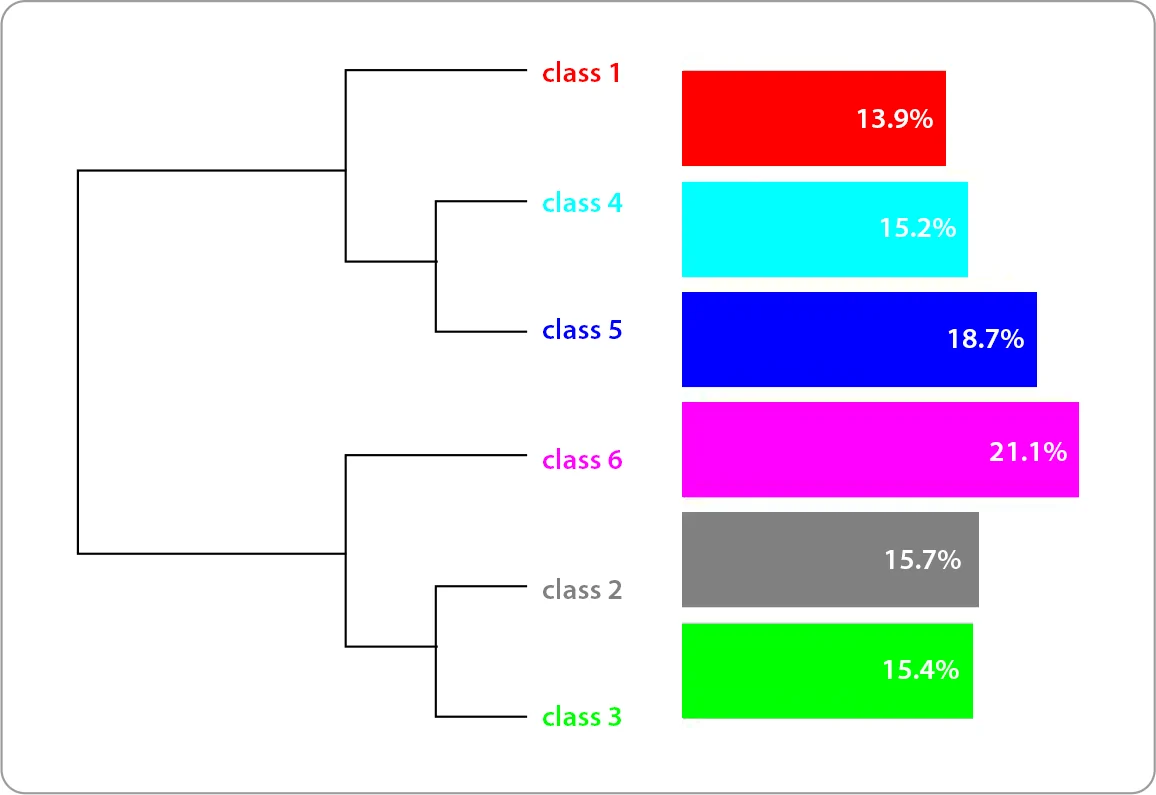
Dendrogram of classes based on the textual corpus of the Family Health Strategy team, Recife, Pernambuco, Brazil, 2023

*Source: IRAMUTEQ 0.7 alpha 2, 2014.*

In the analysis of the textual corpus using DHC, 80.33% of the initial corpus was retained. These results are displayed in a dendrogram consisting of six classes, formed based on the vocabulary and shared meanings identified in the textual analysis (Figure 1).

**Figure 2:**
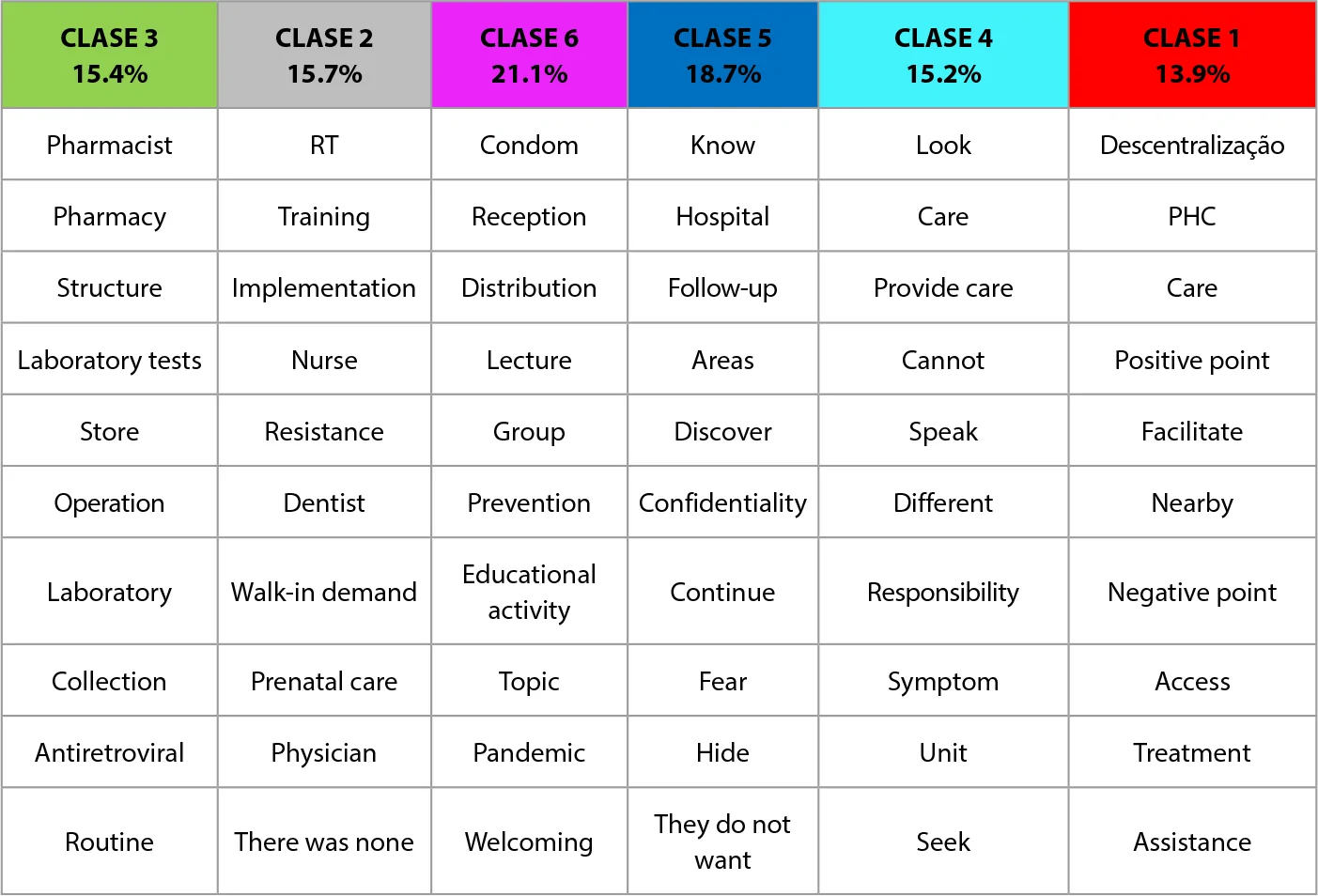
Dendrogram showing the distribution of vocabulary across classes, according to Descending Hierarchical Classification of the Family Health Strategy team, Recife, Pernambuco, Brazil, 2023

The following words showed the highest frequency in the textual corpus: Rapid Test (RT), PHC, thus, know, care, perform, SAE, come, nurse, room, condom, hospital, decentralization, prevention, positive point, refer, prejudice (Figure 2).

*RT - Rapid Test; PHC - Primary Health Care.*

*Source: Adapted. IRAMUTEQ 0.7 alpha 2, 2014.*

Based on the analysis of the most frequent words identified in the dendrogram, titles were assigned to each class as follows: (1) Tensions and Mediations in the Decentralization of Care for PLHIV in PHC; (2) Training Mediations: Capacity Building as an Actor in Enhancing Care; (3) Weakened Support Networks: The Role of Pharmaceutical Services; (4) PHC as the Coordinating Hub of Network-Based Care; (5) Controversies in Continuity of Care: Fear, Confidentiality, and Exposure; and (6) Activation of Health Promotion, Prevention, and Surveillance within the Care Network

### Class 1: Tensions and mediations in the decentralization of care for PLHIV in PHC

Overall, Class 1 showed that FHS professionals recognized both the strengths and weaknesses of care provided to PLHIV in PHC. In this context, they acknowledged the importance of decentralizing care; however, they pointed to barriers related to the work process, fragile care flows, poor physical infrastructure in health units, and team workload overload.

[...] *For decentralization to be implemented, a very large movement would have to take place, with changes in flows, greater management support, and, in fact, an important restructuring of several PHC axes.* (P03)

*I don’t know whether we would be able to provide routine tests; we don’t collect these laboratory tests here at the health unit. That’s why, when the patient is diagnosed, the physician already refers them to the SAE.* (NT17)

[...] *Each level of complexity has its importance, its role, and its responsibilities. Placing an HIV patient in PHC, which relies on low-intensity technologies, is something to reflect on. We don’t use high-technology resources or have specialized professionals. I believe there is a lack of understanding regarding the responsibilities and the role of each level of complexity.* (P8)

### Class 2: Training mediations-training as an actor in improving care

In Class 2, special emphasis was placed on performing the Rapid Test as a strategy for detecting HIV/AIDS. All team professionals indicated the need for technical training focused on test performance and for strengthening dissemination of existing referral protocols, according to diagnostic outcomes, treatment, and clinical management.

Another relevant point concerned the workload associated with performing the RT, viewed as an additional task for the team. In this regard, nurses were seen as bearing the greatest responsibility for RT performance.

*Here at the unit, no other professional wanted to take on the rapid test. Only the nurse was responsible for the rapid test. The other professional categories report that, because it is a high-risk procedure, there should be an incentive-an additional payment.* (N12)

[...] *multiprofessional care, a structured pharmacy, confidentiality, welcoming, time, professional availability, and training for this professional to provide this kind of care. PHC still does not have the physical infrastructure, training, laboratory, or pharmacy to address the complexity of PLHIV care.* (N07)

### Class 3: Weakened support networks-the role of pharmaceutical services

Class 3 addresses, within the theme of service structure and workflow, pharmaceutical services as a potentially strong pillar of healthcare. Among team members’ accounts, nursing technicians highlighted limited physical space and inadequate conditions for storing additional medications, staff shortages for dispensing and counseling, and, notably, the absence of pharmacists in health units.

*The pharmacy structure is complicated, because there are medications that sometimes require a refrigerator, if they need to be kept refrigerated. In the pharmacy, there are administrative staff; we don’t have a pharmacist.* (NT4)

*The unit does not have the structure to provide HIV care; the laboratory test service is not working. There is no pharmacy or pharmacist; the person who could provide guidance about the medication-if this were implemented-would, I believe, be the whole FHS team and, specifically, the physician at the first prescription.* (NT5)

*The patient with HIV has their care, treatment, and follow-up provided by the SAE health team. In the SAE, there are trained and specialized professionals in PLHIV care. PHC has this first contact, welcoming, and identification, because we are generalists. Therefore, I understand that the best pathway for PLHIV must continue in the SAE and not in PHC.* (P2)

*I think the pharmacy does not have the structure to store antiretrovirals; there is no pharmacist, but we do have NASF coverage.* (CHW09)

### Class 4: PHC as the coordinating axis of networked care

Class 4 addressed the process of discovering HIV/AIDS, the initial impact of diagnosis, and the importance of the actors involved in healthcare (family, professionals, managers, and the community).

*I think decentralizing care to PHC is interesting, because it enables patient access. Often, the patient lives far from the reference units* [SAE]*. This change would certainly facilitate patient access within the Health Care Network.* [...] *Healthcare goes beyond treatment and beyond diagnosis, in a very continuous process, with a lot of guidance, education, and health, in fact.* (N47)

*I think this care proposal is very good! It’s important that we are always up to date. I think it would make patients’ lives much easier. We notice that the patient themselves often has some resistance to saying they have the disease, because of fear, prejudice, or something like that; so the PLHIV themselves would be a barrier to their own care.* (N43)

*Decentralization is an interesting proposal. But, honestly, I see more difficulties than facilitation for patients and professionals. Each case deserves care according to its specificity. And, in these cases, care should indeed take place in the SAE, as has always happened. Treatment requires a specialized perspective that generalists do not have.* (P10)

### Class 5: Controversies in continued care: fear, confidentiality, and exposure

Class 5 also points to stigma and prejudice as factors supporting continued treatment in the SAE. Exposure of the diagnosis within the territory becomes a possibility (since PHC is located in the territory), in addition to the technical capacity of professionals in specialized services.

Physicians stated, almost unanimously, that users’ denial of the disease stems from fear of exposure (as a difficulty for adherence within PHC) and from the expertise of SAE professionals, factors supporting continuity of care for PLHIV at secondary and tertiary levels.

*Another fact that needs to be remembered is that many PLHIV do not want to be exposed, because of fear, prejudice, and stigma.* (P03)

*We are able to provide support in our area, and these people are well assisted in our unit-the closest to them! Patient care in PHC does facilitate it, for sure. Prejudice is the main barrier to this care.* (NT12)

*So, you see this issue of exposure in the community as perhaps a possible barrier that would exist for their care and adherence at the health unit.* (N08)

*A negative point would be the issue of confidentiality, especially because we work with people from the community itself.* (N04)

[...] *The complexity of a patient with an HIV diagnosis would have as its main barrier the exposure that generates prejudice, which is still very present for patients who have the virus* [...]. (N02)

### Class 6: Activation of health promotion, prevention, and surveillance in the care network

Finally, Class 6 focused on actions and activities aimed at strengthening health promotion, disease and harm prevention, and health surveillance. Thus, educational actions, home visits, lectures, guidance, and campaigns are strong proposals for behavior change and for disseminating knowledge among health service users. This class was primarily represented by the CHW category as members of the health team.

*Health education activities related to STIs and HIV are carried out through lectures by CHWs in the waiting room, when the patient is waiting for care or for an exam to be entered into the system. We take advantage of the moment and talk to patients. There are also thematic months, such as Purple January, Pink October, Blue November, when we carry out a large health action.* (CHW01)

*We work with guidance and prevention; I think that helps a lot, and when people follow the guidance we give, it prevents them from getting certain diseases, for example STIs-we provide guidance and offer condoms.* (CHW04)

*The Rapid Test* [RT] *was provided through health actions. Only the nurse on our team did it. The other professionals refused to perform the RT.* (CHW04)

## DISCUSSION

Among professionals, different positions and roles were observed regarding the decentralization process. Nurses occupied a central role in performing rapid testing (RT), assuming increasing responsibilities in response to the resistance of other professional categories to adopt new practices. Nursing technicians, in turn, pointed out weaknesses in pharmaceutical services, emphasizing the importance of adequate infrastructure and the qualified presence of pharmacists as essential components of the care network.

Physicians demonstrated a strong attachment to the specialized-care model, expressing doubts about the problem-solving capacity of Primary Health Care (PHC) in relation to HIV, particularly concerning confidentiality and clinical expertise. In contrast, Community Health Workers (CHWs) identified educational activities as a potential means to strengthen bonds and promote health.

These sometimes conflicting perspectives should not be viewed as obstacles but rather as legitimate expressions of a dynamic and evolving network. Actor-Network Theory (ANT) suggests that controversies, instabilities, and resistance are productive forces capable of generating new arrangements and care practices more attuned to local contexts^(1,3)^.

In comprehensive care for people living with HIV (PLHIV), human actors include managers, Family Health Strategy (FHS) and specialized-service professionals, researchers, users, family members, and the community. Non-human elements include physical infrastructure (health units and SAE), laboratories, pharmacies, supplies, medications, tests, and technologies. Connections between these human and non-human elements shape a complex network that sustains comprehensive care across different levels of care^(1,8,15,16)^.

Historically, care for PLHIV has been centered in specialized services, which received the main resources and training, legitimizing themselves as spaces of technical and symbolic dominance in care^(7)^. Currently, decentralizing care means promoting shared care and bringing individuals closer to the care network. It is a challenging process that requires investments, incentives, mapping of services, flows, weaknesses, and successful practices.

The decentralization of care for People Living with HIV (PLHIV) from specialized services to Primary Health Care represents a strategic effort to reorganize the healthcare network, expand access, and enhance the quality of care. This process involves recognizing PHC as a legitimate and collectively constructed space of care.

For nurses, sharing responsibilities between Specialized Care Services (SAE) and PHC constitutes a significant advancement. However, they highlight the work overload that arises from the concentration of responsibilities within their role. Physicians and Community Health Workers also acknowledged this perception. The implementation of the new model encountered resistance from physicians and dentists, who declined to assume responsibilities related to rapid testing (RT), generating tension among team members. Disagreements were also reported among nurses themselves regarding test performance.

The combination of nursing overload and interpersonal conflicts undermines the consolidation of decentralization and compromises the overall work process^(7,17-20)^. From an ANT perspective, resistance and the shifting of responsibility generate controversies and productive tensions that drive adjustments and change^(1,3)^.

For service users, HIV testing remains predominantly targeted to specific groups-such as pregnant women and individuals with tuberculosis-thereby reinforcing inequalities in access within the same territory. Nursing technicians emphasized the critical role of pharmaceutical services, recognizing the pharmacy as an essential link in achieving therapeutic success, given the symbolic centrality of antiretroviral therapy (ART).

Nevertheless, low adherence to ART reflects risk behaviors and may compromise infection control by increasing transmission rates and treatment failures. This, in turn, can lead to the need for more complex drug regimens and a higher potential for adverse effects^(21)^.

From the physicians’ perspective, care for PLHIV should remain at secondary and tertiary levels of complexity, as specialists in Specialized Care Services (SAE) and/or reference hospitals are believed to deliver more accurate and resolutive diagnostic and therapeutic decisions. Given that PHC is the main entry point to the network and Family Health Strategy (FHS) physicians are general practitioners, their role in care is understood as identification, initial assessment, and referral of these users within the Health Care Network (HCN).

The recognition of FHS physicians as generalists reinforces the importance of technical capacity building and effective management to ensure professional confidence and safety in leading care pathways for this population group. Consequently, these professionals considered that treatment management-both from the user and provider standpoint-should remain centralized in the SAE^(22,23)^.

Because they are generalists, FHS physicians believe follow-up of PLHIV should remain the responsibility of specialized services, with greater diagnostic and therapeutic capacity^(22,23)^. They also expressed concern about user confidentiality, given the risk of exposure in units located within the territories themselves.

By proposing the capillarization of care, decentralization generates conflicts that act as engines for transformation and updating of processes. Social actors are fundamental in this movement of negotiation, tension, and reconfiguration^(1,3,16)^.

While physicians highlighted the risk of stigmatization associated with territorial proximity, CHWs viewed this characteristic as an advantage for building bonds with users. As community members, they become trusted figures who promote educational actions and facilitate dialogue^(24)^. CHW activities strengthen health promotion and prevention and contribute to addressing social dilemmas related to HIV^(2)^. From an ANT perspective, their symbolic and practical role directly influences the expansion of care and access^(15,16)^. Another point worth emphasizing is that CHW professional practice is oriented toward health-related activities, with a focus on health promotion and disease prevention. Taken together, these factors underscore their importance as human actors who enhance effective communication between the health team and community members^(24)^.

Within the Family Health Strategy, the operationalization of decentralization requires the re-signification of clinical practices, power relations, and bodies of knowledge. These changes generate tensions but also create opportunities to transform entrenched practices^(1,3,7)^. Thus, understanding the work process from the perspective of FHS professionals is essential to advancing the decentralization of care for People Living with HIV (PLHIV). This understanding can guide public management in developing policies that are better aligned with territorial realities^(1,8)^.

International studies indicate that, following the decentralization of care to Primary Health Care (PHC), most PLHIV are willing to initiate ART, with increased access to services associated with reduced direct costs and closer supervision and interaction between providers and clients. In this sense, decentralization emerges as a key strategy for increasing user satisfaction and improving adherence to health services^(25-28)^.

### Study limitations

This study was conducted in a single municipality in the state of Pernambuco, which limits the generalizability of its findings. Further research employing different methodological approaches and carried out in diverse contexts is recommended to deepen the understanding of the decentralization of care for People Living with HIV (PLHIV) within PHC.

### Contributions to the nursing field

The findings contribute to identifying strengths and weaknesses in the care of People Living with HIV (PLHIV), supporting more effective planning that can positively impact both patients’ quality of life and the organization of work processes. The territorial proximity of health units emerged as a key facilitator for access, particularly for users facing mobility or transportation barriers. For FHS professionals, these results provided insight into concrete strategies and challenges involved in delivering care within local territories.

## FINAL CONSIDERATIONS

Considering the understanding of comprehensive care for People Living with HIV (PLHIV) provided by the FHS within the process of decentralizing services to PHC, it can be stated that HIV permeates the interconnections among care dimensions and the relationships established between network actors and different levels of complexity. These associations and interactions are intrinsically linked to the broader economic, political, cultural, and social context.

From the nurses’ perspective, the category emphasized the overload and accountability placed on these professionals in performing rapid testing. Nursing technicians identified barriers in the pharmaceutical services workflow as key factors that weaken PLHIV care within PHC.

Physicians underscored the importance of maintaining specialized services capable of providing more targeted care to patients. For CHWs, educational practices directed toward the population represent the main driver for disseminating knowledge and promoting behavioral and attitudinal change.

Overall, in the team’s perception, the territorial proximity of health units was identified as a strength, contributing to increased coverage of rapid testing and ART. Conversely, the main weaknesses included the need for enhanced team training and the assurance of confidentiality and privacy in case management within health units. Another limitation identified was the shifting of responsibilities, whether in performing rapid testing at the unit level or referring users to higher levels of care complexity.

## Data Availability

Not applicable.
