# Supplementary material for: Family Health Strategy Team’s Understanding of HIV: Actor-Network Theory
Source: Rev Bras Enferm. 2026 Aug 21;79:e20250313. doi: 10.1590/0034-7167-2025-0313 (PMC13505565; doi:10.1590/0034-7167-2025-0313)
Supplement: Translation

# Translation: Compreensão da Equipe da Estratégia Saúde da Família acerca do HIV: Teoria Ator-Rede

**DOI:** 10.1590/0034-7167-2025-0313pt

**Authors:** Morgana Cristina Leôncio de Lima, Mônica Alice Santos da Silva, Clarissa Mourão Pinho, Luciana da Rocha Cabral, Erika Simone Galvão Pinto, Tatiane Gomes Guedes, Jael Maria de Aquino, Maria Sandra Andrade

**Affiliations:** I Universidade de Pernambuco. Recife, Pernambuco, Brasil; II Universidade Federal do Rio Grande do Norte. Natal, Rio Grande do Norte, Brasil; III Universidade Federal de Pernambuco. Recife, Pernambuco, Brasil

**Keywords:** Soropositividade para HIV, Atenção Primária à Saúde, Assistência Integral à Saúde, Estratégias de Saúde Nacionais, Enfermagem de Saúde Pública.

## Abstract

**Objetivos:**

compreender o cuidado em saúde às pessoas vivendo com HIV, sob a ótica dos profissionais da Estratégia Saúde da Família, à luz da Teoria Ator-Rede.

**Métodos:**

pesquisa qualitativa com profissionais da Estratégia Saúde da Família de Recife, Brasil. Os dados foram coletados entre outubro de 2022 e abril de 2023, mediante entrevistas semiestruturadas. A análise ocorreu com auxílio do software IRAMUTEQ, utilizando a Classificação Hierárquica Descendente.

**Resultados:**

emergiram seis categorias temáticas - mediações formativas: capacitação como ator na qualificação do cuidado; redes de suporte fragilizada: o papel da assistência farmacêutica; Atenção Primária à Saúde como eixo articulador do cuidado em rede; controvérsias no cuidado continuado: medo, sigilo e exposição; e ativações da promoção, prevenção e vigilância em saúde na rede de cuidado.

**Considerações Finais:**

destacam-se desafios como a universalização do teste rápido, resistência de algumas categorias profissionais às novas atribuições, manutenção do sigilo diagnóstico e persistência do estigma.

## INTRODUÇÃO

A descentralização do cuidado às Pessoas Vivendo com HIV (PVHIV) para a Atenção Primária à Saúde (APS), sob a ótica dos profissionais de saúde da equipe mínima da Estratégia Saúde de Família (eSF) e ancorado na Teoria Ator-Rede (TAR), revela um processo complexo de reorganização das práticas, saberes e relações entre múltiplos atores - humanos e não humanos - que compõem a rede de cuidado^(1,2)^. A compreensão da equipe da Estratégia Saúde da Família (ESF) evidencia que esse movimento não se limita à incorporação de novas atribuições, mas implica na reconfiguração das redes de cuidado existentes, provocando instabilidades, resistências e negociações que são próprias de qualquer transformação significativa nos modos de cuidar^(1,3)^.

Apesar dos avanços terapêuticos, ainda se observa o aumento no número de novas infecções pelo HIV. Nesse contexto, o fortalecimento da atuação de equipes multiprofissionais pode favorecer uma abordagem mais enfática na promoção da saúde. Nessa perspectiva, em 2014, teve início, no cenário nacional, a recomendação de reorganização do modelo assistencial voltado às PVHIV. A descentralização para a Atenção Primária à Saúde (APS) passou a ser considerada elemento estratégico para coordenação do cuidado^(4)^.

Nesse sentido, é fundamental reconhecer a singularidade, a diversidade e as especificidades de cada território. A combinação dos saberes dos diferentes profissionais na construção de um cuidado articulado, ampliado e dialógico remete a um modelo de atenção baseado no “território vivo”, com ênfase no trabalho de equipes multiprofissionais e com orientação comunitária^(5,6)^.

A articulação entre os elementos humanos (profissionais, usuários, gestores) e não humanos (testes rápidos, estruturas físicas, protocolos, sistemas de informação) é o que possibilita - ou fragiliza - a efetivação do cuidado integral. Portanto, o êxito da descentralização dependerá da capacidade de reconhecer e mobilizar todos os atores da rede, de modo a promover diálogo, pactuação e corresponsabilidade^(2,7)^.

Avançar na consolidação da APS como espaço legítimo do cuidado às PVHIV exige mais do que diretrizes normativas, requer escuta atenta às redes em movimento, sensibilidade às controvérsias e investimento na estabilização dos elos frágeis do sistema. É nesse processo de composição contínua entre saberes, práticas e tecnologias que se constrói um cuidado verdadeiramente integral e compartilhado^(1,8)^.

## OBJETIVOS

Compreender o cuidado em saúde às pessoas vivendo com HIV, sob a ótica dos profissionais da Estratégia Saúde da Família, à luz da Teoria Ator-Rede.

## MÉTODOS

### Aspectos éticos

Esta pesquisa respeitou as Diretrizes e Normas de Pesquisa Envolvendo Seres Humanos, conforme a Resolução 466/12 do Conselho Nacional de Saúde (CNS). Os participantes assinaram o Termo de Consentimento Livre e Esclarecido e o Termo de Autorização de Gravação de Voz. Foram asseguradas a privacidade e a proteção das informações coletadas, com a aprovação prévia do Comitê de Ética em Pesquisa sob parecer de número: 60697422.4.0000.5192.

Para assegurar o anonimato dos participantes, as entrevistas foram codificadas de forma alfanumérica, identificando-se as falas pelas iniciais da categoria profissional, quais sejam: E (Enfermeiro), T (Técnico de Enfermagem) M (Médicos) e A (Agentes Comunitários de Saúde), seguidas por números sequenciais.

### Referencial teórico-metodológico

A análise dos dados foi ancorada na TAR, de Bruno Latour, que concebe os fenômenos como associações entre atores humanos e não humanos interconectados em redes^(1,8)^. O referencial teórico possibilita compreender os fluxos dos serviços de saúde, as interações e mediações que constituem as práticas, políticas e transformações nas redes de atenção^(2)^.

### Tipo de estudo e Procedimentos metodológicos

Trata-se de um estudo exploratório, com abordagem qualitativa, que buscou compreender o cuidado em saúde prestado às PVHIV atendidas na APS. Seguiram-se os critérios do *Consolidated Criteria for Reporting Qualitative Research* (COREQ).

### Cenário de estudo

O estudo foi desenvolvido na rede de APS do município do Recife, Pernambuco, cuja cidade é organizada em oito Distritos Sanitários (DS), com 133 Unidades de Saúde da Família (USF) e 281 equipes de Saúde da Família^(9)^.

Em relação aos serviços especializados para PVHIV sob gerência municipal, Recife conta com dois Serviços de Assistência Especializada (SAE): o primeiro localizado na Policlínica Lessa de Andrade (DS IV) e o segundo, na Policlínica Gouveia de Barros (DS I), que também abriga o SAE pediátrico e o Centro de Testagem e Aconselhamento (CTA)^(9)^. Há, ainda, o CTA, na Policlínica Salomão Kelner (DS II). Na atenção terciária, destacam-se o Hospital Correia Picanço, o Hospital das Clínicas e o Hospital Universitário Oswaldo Cruz.

### Participantes do estudo

Participaram do estudo profissionais de saúde da equipe mínima da eSF composta por médico, enfermeiro, auxiliar ou técnico de enfermagem e Agente Comunitário de Saúde (ACS)^(10)^. Foram critérios de inclusão: profissionais de ambos os sexos, efetivos ou contratados, médicos, exercendo funções assistenciais nas USF estudadas, e com, pelo menos, um ano na unidade. Excluiu-se: profissionais afastados por licença médica, maternidade ou outras razões superiores a 90 dias.

A seleção das unidades foi realizada por conveniência, e as entrevistas ocorreram nos estabelecimentos de saúde ou em local indicado pelo participante. A definição do número de entrevistas seguiu o critério de saturação teórica, considerado atingido quando novas informações não emergiam nas falas por categoria profissional^(11)^. Houve uma recusa, justificada pela indisponibilidade de tempo do participante.

### Coleta e organização dos dados

Os dados foram coletados entre outubro de 2022 e abril de 2023, por meio de entrevistas semiestruturadas individuais, agendadas previamente por telefone ou presencialmente. As entrevistas foram realizadas durante a jornada de trabalho dos participantes, em ambiente reservado nas unidades de saúde.

O instrumento utilizado foi um roteiro de entrevista que abordava aspectos objetivos e subjetivos do cuidado às PVHIV, abrangendo temas como acesso, diagnóstico, estrutura dos serviços, capacitações, ações de prevenção e educação em saúde.

### Etapas do trabalho

A análise dos dados seguiu a metodologia de análise temática de conteúdo^(11)^, estruturada em três fases: 1) pré-análise, 2) exploração do material 3) tratamento dos resultados e interpretação dos dados. As entrevistas foram transcritas integralmente para posterior análise^(11)^.

### Análise dos dados

O corpus textual das entrevistas foi analisado com o auxílio do software *Interface de R pour les Analyses Multidimensionnelles de Textes et de Questionnaires* (IRAMUTEQ), utilizando-se da técnica de Classificação Hierárquica Descendente (CHD). A CHD organiza o material textual com base na proximidade léxica e de ideias, gerando dendrogramas que ilustram a estrutura das classes temáticas^(12-14)^.

A interpretação crítica dos dados foi realizada à luz da Teoria Ator-Rede^(1,8)^, que permite leitura reflexiva das relações e controvérsias identificadas no processo de descentralização do cuidado às PVHIV.

## RESULTADOS

Participaram do estudo 89 profissionais das USF, sendo 25 enfermeiros, 21 técnicos de enfermagem, 12 médicos e 31 Agentes Comunitários de Saúde. Do total, 73 (82%) eram do sexo feminino, com idades variando entre 20 anos e 65 anos, sendo a média de 44 anos. Quanto ao estado civil, 38 participantes (42,7%) declararam-se solteiros, percentual muito próximo ao dos que afirmaram ser casados (41,6%).

Em relação à análise do corpus textual realizada por meio da Classificação Hierárquica Descendente (CHD), obteve-se aproveitamento de 80,33% do corpus inicial. O resultado da CHD está representado em um dendrograma, composto por seis classes, formadas de acordo com os vocábulos e respectivos sentidos extraídos da análise textual (Figura 1).

**Figura 1:** Dendograma das classes, a partir do corpus textual da Equipe de Estratégia Saúde da Família, Recife, Pernambuco, Brasil, 2023

*Fonte: IRAMUTEQ 0.7 alpha 2, 2014.*

As seguintes palavras obtiveram maior frequência no corpus textual: Teste Rápido (TR), APS, assim, saber, atendimento, realizar, SAE, vir, enfermeiro, sala, preservativo, hospital, descentralização, prevenção, ponto positivo, encaminhar, preconceito (Figura 2).

**Figura 2:** Dendograma referente à distribuição do vocabulário das classes, segundo a Classificação Hierárquica Descendente da Equipe da Estratégia Saúde da Família, Recife Pernambuco, Brasil, 2023

*TR - Teste Rápido; APS - Atenção Primária em Saúde. Fonte: Adaptado. IRAMUTEQ 0.7 alpha 2, 2014.*

A partir da análise das palavras mais frequentes apontadas do dendograma, atribuiu-se diferentes títulos a cada classe distribuída no dendograma, conforme apresentado: 1) Tensionamento e mediações na descentralização do cuidado à PVHIV na APS; 2) Mediações formativas: capacitação como ator na qualificação do cuidado; 3) Redes de suporte fragilizada: o papel da assistência farmacêutica; 4) A APS como eixo articulador do cuidado em rede; 5) Controvérsias no cuidado continuado: medo, sigilo e exposição; e 6) Ativações da promoção, prevenção e vigilância em saúde na rede de cuidado.

### Classe 1: Tensionamento e mediações na descentralização do cuidado à PVHIV na APS

No geral, observou-se, na Classe 1, que os profissionais da equipe da ESF compreendiam as potencialidades e fragilidades do atendimento às PVHIV na APS. Nesse ínterim, reconheceram a importância da descentralização do atendimento, contudo, apontaram como entraves as dificuldades relacionadas ao processo de trabalho, fluxos assistenciais frágeis, estruturas físicas precárias das unidades de saúde e sobrecarga dos profissionais da equipe.

[...] *Para efetivação da descentralização, teria que acontecer um movimento muito grande com mudanças de fluxos, mais suporte da gestão e, de fato, uma reestruturação importante de vários eixos da APS.* (M 03)

*Eu não sei se teria condições de fornecer os exames de rotina, a gente não coleta esse exame laboratorial aqui na unidade de saúde. É tanto que quando o paciente é diagnosticado, o médico já encaminha para o SAE.* (T17)

[...] *Cada nível de complexidade tem sua importância, seu papel e suas atribuições. Colocar o paciente HIV na APS que tem tecnologia leve é algo para refletir. Não temos uso de alta tecnologia e profissionais com especialidade. Creio que está havendo uma falta de entendimento das atribuições e do papel de cada nível de complexidade.* (M8)

### Classe 2- Mediações formativas: capacitação como ator na qualificação do cuidado

Na Classe 2, obteve-se enfoque especial na execução do TR como estratégia de detecção do HIV/Aids. Sinaliza-se que todos os profissionais da equipe solicitaram capacitações técnicas voltadas para execução dos testes e fortalecimento da divulgação dos protocolos existentes de encaminhamentos e assistências, conforme os desfechos diagnósticos, tratamento e condução clínica.

Outro ponto relevante esteve relacionado à sobrecarga causada pela execução do TR, sendo atribuída como mais uma atividade para a equipe. Para esse ponto, atribuindo-se à categoria dos enfermeiros a maior responsabilização nessa execução do TR.

*Aqui, na unidade, nenhum outro profissional quis aderir ao teste rápido. Só o enfermeiro ficou responsável pelo teste rápido. As outras categorias profissionais relatam que por ser um procedimento de risco, devia haver um incentivo. Uma gratificação.* (E12)

[...] *atendimento multiprofissional, farmácia estruturada, sigilo, acolhimento, tempo, disponibilidade do profissional, capacitação desse profissional para estar fornecendo esse tipo de atendimento. A APS ainda não tem estrutura física nem de capacitação, nem de laboratório, nem de farmácia, para estar atendendo à complexidade que é o PVHIV.* (E 07)

### Classe 3: Redes de suporte fragilizada: o papel da assistência farmacêutica

A Classe 3 aborda, dentro da temática, a estrutura dos serviços e do fluxo do processo de trabalho, a assistência farmacêutica, como pilar potente para o cuidado em saúde. Destaca-se que entre as falas dos membros da equipe, os técnicos de enfermagem apontaram o espaço físico como pequeno e sem condições de armazenamento de mais medicamentos, déficit de profissionais para dispensação e orientações, com ênfase para ausência de farmacêuticos nas unidades de saúde.

*A estrutura da farmácia é complicada, porque aqui tem medicações que, às vezes, não tem geladeira, se for o caso de medicação que precise ficar refrigerada. Na farmácia, têm pessoas do administrativo, não temos farmacêutico.* (T4)

*A unidade não possui estrutura para realização do atendimento ao HIV, o exame laboratorial não está funcionando. Não existe farmácia nem farmacêutico, quem poderia dar as orientações sobre o medicamento, caso houvesse essa implantação, creio que toda a equipe da ESF e, em específico, o médico na primeira prescrição.* (T5)

*O paciente com HIV tem seu atendimento, tratamento e acompanhamento pela equipe de saúde do SAE. No SAE, existem profissionais capacitados e especializados no cuidado à PVHIV. A APS tem esse primeiro contato, acolhimento e identificação, por sermos generalistas. Logo, entendo que o melhor fluxo para as PVHIV tem que continuar no SAE e não na APS.* (M2)

*Acho que a farmácia não tem estrutura para armazenar os antirretrovirais, não tem um farmacêutico, mas temos a cobertura do NASF.* (ACS 09)

### Classe 4: APS como eixo articulador do cuidado em rede

A Classe 4 abordou o processo de descoberta do HIV/Aids e o impacto inicial do diagnóstico e da importância dos atores envolvidos no cuidado em saúde (família, profissionais, gestores e comunidade).

*Eu acho que descentralizar o atendimento para APS é interessante, até porque viabiliza a questão do acesso ao paciente. Muitas vezes, o paciente mora longe das unidades de referência* [SAE]*. Com certeza, essa mudança facilitaria o acesso do paciente na RAS.* [...] *O cuidado em saúde vai além do tratamento, além do diagnóstico, em um processo muito contínuo, de muita orientação, educação e saúde, na verdade.* (E47)

*Acho muito bom a proposta desse atendimento! Importante sempre estarmos atualizados. Acho que facilitaria bastante a vida dos pacientes. A gente percebe que o próprio paciente infectado tem certa resistência em dizer que está com a doença, que tem medo, preconceito ou uma coisa assim, então, a própria PVHIV seria uma barreira para o seu atendimento.* (E43)

*A descentralização é uma proposta interessante. Mas, sinceramente, vejo mais dificuldades do que facilidade para os pacientes e profissionais. Cada caso merece o atendimento, de acordo com a especificidade. E, nesses casos, de fato, o atendimento deve ser no SAE, como sempre aconteceu. O tratamento precisa de um olhar especializado que os generalistas não têm.* (M10)

### Classe 5: Controvérsias no cuidado continuado: medo, sigilo e exposição

A Classe 5, também, traz o estigma e preconceito como fatores para corroborar a continuidade do tratamento no SAE. A exposição do diagnóstico dentro do território torna-se uma possibilidade (afinal a APS está dentro do território), além da capacidade técnica dos profissionais dos serviços especializados.

Os médicos apresentaram, de forma quase unânime, entre as entrevistas que a negação da doença por parte dos usuários se deve ao medo da exposição (como dificuldade para adesão na APS) e à expertise dos profissionais dos SAE, fatores para continuidade da assistência às PVHIV nos níveis de atenção secundário e terciário.

*Outro fato que precisa ser lembrado é que muitas PVHIV não querem ser expostas, por medo, preconceito e estigma.* (M 03)

*A gente consegue dar suporte na nossa área e esse pessoal é bem assistido na nossa unidade. O mais próximo deles! O atendimento dos pacientes na APS facilita sim, com certeza. O preconceito é a principal barreira para esse atendimento.* (T12)

*Então, você vê essa questão da exposição na comunidade como talvez uma possível barreira que iria existir para o atendimento e adesão dele na unidade saúde.* (E 08)

*Um ponto negativo seria a questão do sigilo até porque nós trabalhamos com as pessoas da própria comunidade.* (E 04)

[...] *A complexidade que é um paciente que tem um diagnóstico de HIV teria como principal barreira a exposição que gera o preconceito, ainda muito presente com o paciente que tem o vírus* [...]. (E 02)

### Classe 6: Ativações da promoção, prevenção e vigilância em saúde na rede de cuidado

Por fim, a Classe 6 teve como assunto central as ações e atividades voltadas ao fortalecimento da promoção da saúde, prevenção de doenças e agravos e vigilância em saúde. Deste modo, ações educativas, visitas domiciliares, palestras, orientações e campanhas são propostas potentes para mudança de comportamento e disseminação de conhecimento para os usuários de saúde. Esta classe teve como principal representação a categoria dos ACS enquanto membros da equipe de saúde.

*As atividades de educação em saúde relacionadas às IST e HIV são feitas por palestras pelos ACS na sala de espera, quando o paciente está esperando algum atendimento ou colocar algum exame no sistema. Aproveitamos o momento e conversamos com os pacientes. Também, têm os meses temáticos, como janeiro roxo, outubro rosa, novembro azul que fazemos uma grande ação de saúde.* (ACS 01)

*A gente trabalha com orientação e prevenção, eu acho que isso ajuda muito, e quando as pessoas seguem as orientações que damos, evita dela ter certas doenças, por exemplo: IST, que damos orientação e oferecemos preservativo.* (ACS 04)

*O Teste Rápido* [TR] *é fornecido por meio das ações de saúde. Só a enfermeira da nossa equipe que fazia. Houve recusa dos outros profissionais para realizar o TR.* (ACS 4)

## DISCUSSÃO

Entre os profissionais, observou-se diferentes posicionamentos e papéis atribuídos ao processo de descentralização. Os enfermeiros ocupam posição central na execução do TR, acumulando responsabilidades diante da resistência de outras categorias em aderir às novas práticas. Os técnicos de enfermagem, por sua vez, evidenciaram fragilidades da assistência farmacêutica, ressaltando a importância da infraestrutura e presença qualificada de farmacêuticos, como elementos críticos da rede.

A categoria médica demonstrou forte apego ao modelo especializado, colocando em dúvida a capacidade resolutiva da APS frente ao HIV, especialmente no que se refere ao sigilo e à expertise clínica. Em contraste, os ACS identificaram, nas ações educativas, potencialidade para o fortalecimento de vínculos e promoção da saúde.

Essas visões, por vezes conflitantes, não devem ser vistas como entraves, mas como expressões legítimas de uma rede viva em transformação. A TAR ensina que controvérsias, instabilidades e resistências são componentes produtivos, capazes de gerar novos arranjos e formas de cuidado mais alinhadas às realidades locais^(1,3)^.

Na atenção integral às PVHIV, os atores humanos incluem gestores, profissionais da ESF e de serviços especializados, pesquisadores, usuários, familiares e comunidade. Já os elementos não humanos abrangem estruturas físicas (unidades de saúde e SAE), laboratórios, farmácias, insumos, medicamentos, exames e tecnologias. As conexões entre esses elementos humanos e não humanos conformam uma rede complexa que sustenta o cuidado integral nos diferentes níveis de atenção^(1,8,15,16)^.

Historicamente, a assistência às PVHIV esteve centrada nos serviços especializados, que receberam os principais recursos e capacitações, legitimando-se como espaços de domínio técnico e simbólico do cuidado^(7)^. Atualmente, descentralizar o atendimento significa promover cuidado compartilhado e aproximar os indivíduos da rede de atenção. Trata-se de processo desafiador que exige investimentos, incentivos, mapeamento dos serviços, fluxos, fragilidades e práticas exitosas.

A descentralização do cuidado das PVHIV da atenção especializada para a APS representa um movimento estratégico de reorganização da rede, ampliando o acesso e qualificando a assistência. Envolve reconhecer a APS como espaço legítimo de cuidado, construído coletivamente.

Para os enfermeiros, compartilhar o cuidado entre SAE e APS é um avanço importante. No entanto, a categoria denuncia a sobrecarga gerada pela concentração de responsabilidades nessa função. Médicos e ACS entrevistados também reconheceram essa percepção. A implantação do novo modelo encontrou resistência por parte de médicos e dentistas, que se recusaram a assumir atribuições relacionadas ao TR, criando ambiente tenso entre membros das equipes. Inclusive, houve divisão entre os próprios enfermeiros quanto à execução do teste.

A sobrecarga dos enfermeiros e os conflitos interpessoais fragilizam a consolidação da descentralização e comprometem o processo de trabalho^(7,17-20)^. Sob a perspectiva da TAR, as resistências e desresponsabilizações geram controvérsias e tensões produtivas que impulsionam ajustes e mudanças^(1,3)^.

Para os usuários, a testagem ainda é voltada a grupos específicos, como gestantes e pessoas com tuberculose, o que reforça desigualdades no acesso, em um mesmo território. Técnicos de enfermagem destacaram o papel crítico da assistência farmacêutica, reconhecendo a farmácia como elo essencial para o sucesso terapêutico, em razão da centralidade simbólica da TARV.

Contudo, a baixa adesão à TARV é um componente que revela um comportamento de risco, que, de modo geral, pode prejudicar o controle da infecção, ocasionando a ampliação da transmissão e maior número de falência medicamentosa. Como consequência, pode resultar na necessidade de esquemas medicamentosos mais complexos e com maior potencial de efeitos adversos^(21)^.

Na compreensão da categoria médica, a assistência às PVHIV deve ter continuidade nos níveis de complexidades secundário e terciário, pois os especialistas dos SAE e/ou hospital referência terão condutas diagnósticas e tratamentos mais assertivos e resolutivos, visto que a APS é a porta de entrada preferencial da rede, e os profissionais médicos da ESF são generalistas e, no processo de cuidar, tem o papel de captar, identificar e referenciar esses usuários dentro da RAS.

No que diz respeito à competência clínica dos médicos da ESF serem generalistas, corrobora-se a premissa da importância do aprimoramento técnico e de gerenciamento efetivo para garantir a segurança do profissional na condução da linha de cuidado para aquele segmento populacional. Assim, esses profissionais consideraram que tanto para os usuários como para os profissionais, a condução do tratamento deve permanecer centralizada no SAE^(22,23)^.

Médicos da ESF, por serem generalistas, consideram que o seguimento das PVHIV deve permanecer sob responsabilidade dos serviços especializados, com maior capacidade diagnóstica e terapêutica^(22,23)^. Também, manifestaram preocupação com o sigilo dos usuários, considerando o risco de exposição em unidades situadas nos próprios territórios.

A descentralização, ao propor a capilarização do cuidado, gera conflitos que são motores para transformação e atualização dos processos. Os atores sociais são fundamentais nesse movimento de negociação, tensionamento e reconfiguração^(1,3,16)^.

Enquanto os médicos apontaram riscos de estigmatização pela proximidade territorial, os ACS veem nessa característica vantagem para o vínculo com os usuários. Por integrarem a comunidade, tornam-se figuras de confiança, promovendo ações educativas e favorecendo o diálogo^(24)^.

A atuação dos ACS fortalece a promoção e prevenção da saúde, contribuindo para enfrentar os dilemas sociais relacionados ao HIV^(2)^. Sob a lente da TAR, o papel simbólico e prático influencia diretamente na expansão do cuidado e do acesso^(15,16)^. Outro ponto que merece destaque é o foco da atuação profissional do ACS, pautada nas atividades e ações em saúde, com ênfase na promoção da saúde e prevenção de doenças. As somas desses fatores se mostram significativas, isto é, elemento humano que potencializa a boa comunicação entre esse membro da equipe de saúde e os comunitários de saúde^(24)^.

A ESF compreende que instrumentalizar a descentralização exige ressignificar condutas, relações de poder e saberes. As mudanças geram tensões, como também oportunidades de transformação das práticas cristalizadas^(1,3,7)^. Assim, compreender o processo de trabalho, a partir da perspectiva dos profissionais da ESF, é essencial para promover a descentralização do cuidado às PVHIV. Esse entendimento orienta a gestão pública na formulação de políticas mais alinhadas às realidades dos territórios^(1,8)^.

Estudos internacionais apontam que, após a descentralização do cuidado para APS, a maioria das PVHIV mostraram disposição a iniciar a TARV. A expansão do acesso aos serviços está relacionada à diminuição dos gastos diretos, somados à supervisão e interação dos profissionais prestadores dos serviços com os clientes. Portanto, a descentralização proporciona importante alavanca no aumento da satisfação dos usuários, com melhoria na adesão aos serviços^(25,28)^.

### Limitações do estudo

Este estudo foi realizado em apenas um município de Pernambuco, o que limita a generalização dos achados. Recomenda-se a realização de novas pesquisas, com diferentes abordagens metodológicas e em contextos diversos, visando aprofundar a compreensão sobre a descentralização do cuidado às PVHIV, na APS.

### Contribuições para a área da enfermagem

Os resultados contribuem para identificar potencialidades e fragilidades do cuidado às PVHIV, subsidiando planejamento mais eficaz, com impactos na qualidade de vida dos pacientes e na organização do processo de trabalho. A proximidade territorial das unidades se destaca como facilitador do acesso, especialmente para usuários com dificuldades de deslocamento. Para os profissionais da ESF, os achados permitiram reconhecer estratégias e desafios concretos da condução do cuidado em territórios.

## CONSIDERAÇÕES FINAIS

Ao considerar a compreensão do cuidado integral às PVHIV atendidas pela ESF, no processo de descentralização da assistência para a APS, pode-se afirmar que o HIV perpassa pelas conexões das dimensões do cuidar e das relações entre os atores da rede e dos níveis de complexidade da rede. As associações e relações entre os atores e as dimensões do cuidado estão conectadas ao contexto econômico, político, cultural e social.

Na perspectiva dos enfermeiros, a categoria apresentou como reflexão a sobrecarga e responsabilização desses profissionais na execução do teste rápido. Os técnicos de enfermagem apontaram como principais elementos que fragilizam o cuidado da PVHIV, na APS, entraves no processo de trabalho da assistência farmacêutica.

Já a categoria médica abordou a importância e manutenção dos serviços especializados com assistência mais direcionada aos pacientes. Para os Agentes Comunitários de Saúde, o enfoque nas práticas educativas para a população é a mola impulsionadora para disseminar o conhecimento, de modo a permitir a transformação de condutas e atitudes.

No geral, quando se abordou acerca da compreensão da equipe, identificou-se entre as potencialidades a proximidade das unidades dentro do território e, como consequência, o aumento da testagem rápida e da cobertura dos ARV.

Por outro lado, entre as fragilidades, observou-se a necessidade de maior qualificação da equipe e a garantia do sigilo e confidencialidade dos casos identificados na unidade de saúde. Ainda, como fragilidade, é referida a transferência de responsabilidades, seja na execução da testagem rápida, no âmbito na própria unidade, ou no encaminhamento do usuário para outro nível de complexidade.

## Data Availability

Não se aplica.
